# Clinical, Radiological and Pathological Appraisal of Acute Appendicitis in Al Qassim, Saudi Arabia: A Single-Center Retrospective Analysis

**DOI:** 10.7759/cureus.28627

**Published:** 2022-08-31

**Authors:** Fahad Alnuaymah, Amarachukwu Chiduziem Etonyeaku, Hamad S Alsaeed, Abdullah N AlSamani, Atheen A Alshubrmi, Rayan K Aldoubiab, Abdulhakeem A Aloqla, Moath A Almushiqeh

**Affiliations:** 1 General Surgery, Buraidah Central Hospital, Buraidah, SAU; 2 Surgery, Obafemi Awolowo University, Ile-Ife, NGA; 3 College of Medicine, Qassim University, Buraidah, SAU

**Keywords:** acute surgical abdomen, pathology, radiology, clinical features, appendicitis

## Abstract

Background

Acute appendicitis can occur at any age but is rare among people of extreme age; it is more common in teenagers and young adults. Traditionally diagnosis is made on clinical grounds. In recent times imaging techniques have been deployed to improve diagnosis and reduce negative appendicectomy rates. The aim of the study was to describe the common clinical features of acute appendicitis among our patients, highlight the role of medical imaging, and compare all these with the histological report of the excised appendix.

Methods

A 24-months retrospective review of all patients who underwent appendicectomy (July 1, 2019-June 30, 2021) for suspected acute appendicitis was performed. Medical records numbers of patients who had appendicectomies were retrieved from the operating room register. These numbers were used to access the hospital's electronic medical records database for the patients' records. These records were reviewed for biodata, clinical features, laboratory, medical imaging findings, and histological reports.

Result

In this hospital, 354 appendicectomies were performed. Only 336 had complete data set suitable for further review. There were more males (N=257; 76.5%) than females (N=79, 23.5%), yielding a male to female ratio of 4:1. There were also more Saudi citizens (n=266, 79.2%), with the predominant age group being 11-30 years. Abdominal pain was the predominant symptom (100%) and was localized to the right iliac region in 331 (98.7%) of patients. Other symptoms recorded were anorexia (n=247, 73.5%), vomiting (n=190, 56.5%), and nausea (n=93, 27.7%). Atypical symptoms included diarrhoea (n=27, 8%) and constipation (n=12, 3.6%). Acute appendicitis, complicated appendicitis, and no appendicitis were the reported histological disposition in 174 (51.8%), 124 (36.9%), and 38 (11.3%) cases respectively. Abdominal CT scan had a higher sensitivity (98.6% vs 70.5%), higher diagnostic odd ratio (2.5 vs 1.4) and a lower miss (false negative) rate (1.4% vs 29.5%) compared to ultrasonography. However, the CT scan, from this study, has a rather low specificity (3.4%) and high false positive rates (96.5%). Open (n=205; 61%) and laparoscopic (n=131;39%) approaches were used for the appendicectomies. In our study, 44 patients were diagnosed with the decision to operate based on clinical grounds; and of this, 42 (95.4%; n=44) had confirmatory histology reports of appendicitis. Also, 38 patients had negative appendicectomy; giving a negative appendicectomy rate of 11.3%. This high rate may be due to the lower specificity and high false positive rate observed in this study. The post-operative complication rate was 21.4%, and this was solely due to surgical site infection, and this was more common with the open approach (p=0.001).

Conclusion

Suspected acute appendicitis was the sole indication for our appendicectomies. A computerized tomography scan was a more reliable diagnostic tool than ultrasonography. Despite the fact that acute appendicitis is majorly a clinical diagnosis, and good clinical acumen is an excellent skill in the management of patients, we observed an overreliance on medical imaging for diagnosis. Open appendicectomies were more common, and surgical site infection was the sole complication of surgery. There was a relatively high negative appendicectomy rate for an image-assisted diagnosis.

## Introduction

From ancient Egyptian (3100BC-332BC), Greco-Roman (800BC-600AD), medieval (500 to 1400-1500 CE), European renaissance (1400-1700 CE) times and contemporary times, acute appendicitis has remained a major concern for physicians, especially the general surgeon. [1-4]. Over these times, there has been a steady evolution in the diagnosis and treatment of the disease that has been directed at improving disease outcomes. This evolution has seen the shift from diagnosis based purely on clinical features [5, 6], to the inclusion of laboratory investigations [7-9], and lately confirmatory medical imaging techniques [10, 11]. Furthermore, histological assessment of the excised appendix has now become imperative as there are other disease conditions that may mimic acute appendicitis such as mucocele and carcinoid tumor of the appendix, chronic granulomatous infections, actinomycosis, endometriosis, lymphoma, and helminthiasis [12].

Acute appendicitis is the most common cause of abdominal pain requiring emergency surgical intervention [13], and there is a reported 7% lifetime risk for developing appendicitis [14], with the highest incidence happening between the ages of 10 and 30 years [15]. If untreated, acute appendicitis could lead to serious life-threatening complications like appendiceal perforation, abscess formation, and peritonitis [16]. The mortality rate in uncomplicated appendicitis is less than 1% and may reach 5% or more in elderly patients and children. In these latter age groups, the diagnosis of acute appendicitis is often delayed as clinical features may often be vague, with an attendant increased risk of complications [17]. There appears to be a geospatial distribution of the incidence of the disease: with higher rates in low socio-economic climes [18]. This view has been challenged by those who attribute the higher incidence of appendicitis in urban centers to lifestyle changes [19]. Acute appendicitis has been reported as the most common reason for appendicectomy in Northern Saudi Arabia [20]. Evidence shows that intra-operative normal appendices may have an unusual incidental result at pathological evaluation, and the practice of routine pathological examination of appendectomy specimens varies between centers [12, 21].

Although a lot has been written on acute appendicitis in medical literature, the most recent work we could find relating to this subject in our region was done over 15 years ago [22]: before the advent of laparoscopic surgery and modern imaging modalities like computerized tomography (CT) scan.

Thus, this study set out to determine the patients` characteristics, clinical features, radiology assessment and reliability, and histological correlates of the appendix post-appendicectomy in patients managed for acute appendicitis at the Buraidah Central Hospital in the Al Qassim region of the Kingdom of Saudi Arabia.

## Materials and methods

Study design

This is a retrospective descriptive hospital-based study involving the review of patients' electronic medical records for patients who had surgical management of acute appendicitis.

Region of study

The study was carried out in Buraidah Central Hospital, Buraidah, in the province of Al Qassim, Saudi Arabia. This is a 460-bed tertiary hospital in the central part of Saudi Arabia; 80 of these beds are assigned to general surgery services. The patients were managed by the general surgery division comprising: consultants, specialists, and resident doctors.

Study population and sampling

This study involved the purposeful selection of all patients who had appendicectomy for presumed acute appendicitis between July 1, 2019, to June 30, 2021 (24 months) at the Buraidah Central hospital in the Al Qassim region of Saudi Arabia and in whom histological examination of the appendix was done post-surgery.

Our study sample included: all patients who had appendicectomy on account of presumed acute appendicitis, those who had a histological report of the evaluation of their appendix post-surgery, and those patients who had sufficient data (biodata, record of symptoms and clinical signs, laboratory test and clinical course on admission). We exclude those patients who had no record of histological evaluation, and those patients with insufficient records (missing age, gender, clinical features, and course of disease).

Methods for data collection

The operating room operations register was reviewed so as to extract the hospital numbers of all patients who had appendicectomy at the hospital during the study period (July 1, 2019, to June 30, 2021). The hospital numbers so obtained were used to interrogate the hospital`s electronic medical records system (VIDA®) in order to get details of the medical records for each of the patients. Records assessed were for biodata, clinical features at presentation, basic laboratory tests, medical imaging, the clinical course of the disease including interventions, and histopathology appraisal of the excised appendix. The data set obtained was entered into a preformed data spreadsheet.

Research Instrument (Questionnaire) and its Validation

A preformed data sheet was used to extract desired information from VIDA® on the individual patient and this was entered into an Excel® (Microsoft) spreadsheet. The Excel sheet was subsequently exported into the Statistical Package of Social Sciences, SPSS® 23.0 version (IBM Corp., Armonk, NY).

Data Management and Data Analysis

The Excel sheet and the SPSS tool were stored on a personal computer. The records are accessible to all the authors. The data set was analyzed for frequencies, simple percentages, and measures of central tendency (mean, median, and mode) Parametric test using the chi-square test was used to test for the statistical significance of an association.

Ethical consideration

This study complied with the Helsinki declaration and was approved by the Regional Research Ethics Committee of the General Directorate of Health Affairs Al Qassim Region Kingdom of Saudi Arabia: (ethical approval number: 607-43-3972). The patients' identities were kept confidential at all times.

## Results

**Figure 1:**
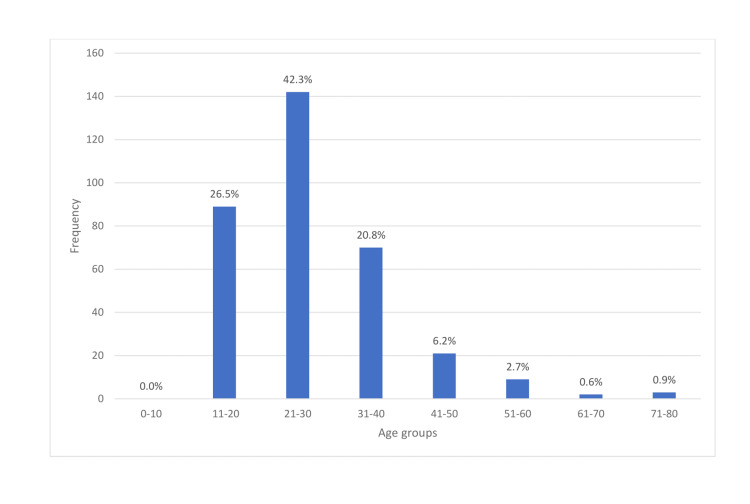
Age group distribution of patients

A total of 354 appendectomies were performed over the 24-month study period (July 2019-June 2021). Only 336 (94.9%) patients had complete data sets suitable for further review, and this report is based on this figure. There were more males (n=257; 76.5%) than females (n=79, 23.5%), yielding a male to female ratio of 3:1. A total of 266 patients (79.2%) were Saudi citizens while the remaining 70 (20.8%) were non-Saudis. The age groups of patients are as shown in Figure 1, with the majority (n=231, 68.8%) experiencing appendicitis in the second and third decades of life irrespective of the gender of the patient. The mean, median and modal age was 27.76 (±10.88), 25, and 24 years respectively. The age ranged from 14-80 years. 

Abdominal pain was the principal complaint in all the patients (n=336; 100%) at presentation and was localized to the right lower abdominal quadrant in 331 (98.5%) of them. Other symptoms recorded were anorexia (n=247, 73.5%), vomiting (n=190, 56.5%), nausea (n=283, 84.2%), diarrhoea (n=27, 8.0%) and constipation (n=12, 3.6%). Around 229 (68.2%) patients presented at the emergency room within 24 hours of the onset of pain, while 85 (25.3%) and 22 (6.6%) presented after 24 hours but within 72 hours, and greater than 72 hours respectively. Most patients (299; 89%) had normal pulse rates while leucocytosis was a common feature in the majority (239; 71.1%) of the patients. Only nine patients (2.7%) had a fever (temperature ≥ 38℃). Table 1 gives further details of common clinical features of the patients.

**Table 1:** Clinical and laboratory characteristics of patients who had appendectomies for acute appendicitis

Characteristics		Frequency	Percent
Duration of pain at presentation	≤ 24 hours	229	68.2
	>24 hours- ≤72 hours	85	25.3
	> 72hours	22	6.5
	Total	336	100
Pulse rate	Normal	299	89
	Tachycardia	37	11
	Total	336	100
White cell count	≤ 11 x 10^9^/L	97	28.9
	> 11- ≤ 20 x 10^9^/L	220	65.5
	> 20 x 10^9^/L	19	5.6
	Total	336	100
Postoperative interval before the commencement of feeds	≤ 24 hours	235	69.9
	>24 hours- ≤ 48 hours	79	23.5
	> 48 hours	22	6.6
	Total	336	100
Post-operative complications (surgical site infection)	None	264	78.6
	Intra-abdominal collection	62	18.4
	Superficial surgical wound infection	10	3.0
	Total	336	100
Duration of hospital stay	≤ 24 hours	29	8.6
	>24 hours- ≤72 hours	220	65.5
	> 72hours	87	25.9
	Total	336	100

Ultrasonography (USS) of the abdomen and pelvis, and contrast-enhanced computerized tomography (CT) scan of the abdomen and pelvis were the two imaging techniques used in this series. The reliability of each of these modalities based on the histological diagnosis is presented in Tables 2-3 respectively.

**Table 2:** Reliability of ultrasound diagnosis relative to histological diagnosis sensitivity = 70.5%, specificity = 36.8%, positive predictive value (PPV) = 88.3%, negative predictive value (NPV) = 15.6%, false negative rate (FNR) = 29.5%, diagnostic odd ratio (DOR) = 1.4

Tests		Histology (standard)		Total
		Positive	Negative	
Ultrasound diagnosis	Positive	91	12	103
	Negative	38	7	45
Total		129	19	148

**Table 3:** Reliability of CT scan diagnosis relative to histological diagnosis sensitivity = 98.6%, specificity = 3.4%, positive predictive value (PPV) = 88.0%, negative predictive value (NPV) = 25%, false negative rate (FNR) = 1.4%, diagnostic odd ration (DOR) = 2.5.

Tests		Histology (standard)		Total
		Positive	Negative	
CT scan diagnosis	Positive	213	28	241
	Negative	3	1	4
Total		216	29	245

The evaluation of the congruency between diagnosis from imaging techniques and histological evaluation of specimen showed that CT was more reliable than ultrasound as it had higher sensitivity (98.6% vs 70.5%), lower false negative rate (1.4% vs 29.5%), and higher diagnostic odd ratio (2.5 vs 1.4). However, in 44 patients (13.1%) diagnosis and decision to operate were based solely on clinical grounds with the patient not having any form of imaging done (Table 4). And of this figure, 42 (95.4%) were confirmed to be appendicitis on histological assessment. A more comprehensive account detailing imaging modalities and histological disposition is presented in Table 4.

**Table 4:** Congruency of imaging diagnosis with histology *Uncomplicated or otherwise called simple appendicitis is the inflammation of the appendix without evidence of necrosis or perforation. **Complicated appendicitis involves transmural inflammation with necrosis and or perforation of the appendix; may be associated with abscess collection

Histology	Medical imaging					Total (N)
	Ultrasound diagnosis		CT diagnosis			
			Positive	Negative	Not done	
*Uncomplicated appendicitis	Positive		28	0	18	46
	Negative		17	0	4	21
	Not done		78	2	27	107
	Total		123	2	49	174
**Complicated appendicitis	Ultrasound diagnosis	Positive	28	0	17	45
		Negative	15	1	1	17
		Not done	47	0	15	62
	Total		90	1	33	124
No appendicitis	Ultrasound diagnosis	Positive	5	0	7	12
		Negative	6	1	0	7
		Not done	17	0	2	19
	Total		28	1	9	38
Grand total			241	4	91	336

All patients had appendectomy using general anesthesia, and the surgical approach is as shown in Table 5.

**Table 5:** Showing surgical approach adopted for appendectomy among patients

Approach	Incision	Frequency	Percent (n=336)
Open surgery (n=205; 61.0%)	Grid iron/Lanz	203	60.4
	Midline infraumbilical	2	0.6
Laparoscopy	Standard 3-port incisions	131	39.0

**Figure 2:**
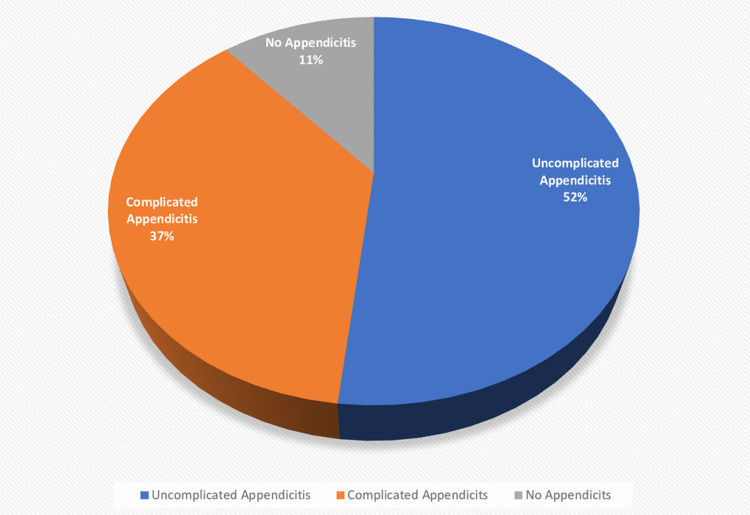
Histological appraisal of appendix specimen

Post-operative complications were the sole surgical site infection (SSI) which was more common in patients who presented late (>24 hours (p=0.000)) and in those who had open surgical technique (p=0.001). Furthermore, patients who had SSI relatively had delays in the commencement of feeds (p=0.000) and a longer hospital stay (p=0.000). A negative appendectomy rate of 11.3% (n=38) was recorded and the disposition of the histological assessment is presented in Figure 2.

## Discussion

There are many indications for appendicectomy, and these include appendicitis, carcinoid tumor, mucocele of the appendix, a graft for urinary tract reconstruction (ileal conduit), and for on-table lavage. Our study supports reports in medical literature indicating that acute appendicitis is the most common indication for appendicectomy [20], while acute appendicitis is the most common indication for emergency abdominal surgery worldwide. Our study also confirmed that acute appendicitis is most common among young individuals, and has male dominance [19, 23]. However, unlike Alahmari et al. [19], most of our patients were Saudi citizens. This may be a function of the population mix in our environment, and the free health services available to citizens while other nationalities either pay out of pocket or access care through medical insurance.

Classical symptoms of acute appendicitis include migratory abdominal pain (umbilicus to the right iliac fossa), anorexia, nausea, and vomiting (often one or two times). These symptoms were common among our patients. Also, some atypical symptoms like diarrhea and constipation were reported by patients, but these were to a lesser degree. Other symptoms and relevant signs for acute appendicitis were either not mentioned, or their documentation was incomplete. Thus, we could not rely upon them and hence were not considered in the analytic process. This underscores the major disadvantage of retrospective studies: where documentation and record keeping may be poor. Acute uncomplicated appendicitis seldom presents with fever, and when it does present with fever, it is often low grade [24]. This also we found to be true. Similarly, the pulse in most of our patients was within normal limits (60-100 beats per minute). Fever (body temperature ≥ 38℃) and tachycardia become prominent in complicated cases like appendiceal perforation, abscesses, and peritonitis (localized or general). The presence of fever and tachycardia may be masked with the use of antipyretics, analgesics, and antibiotics. Due to the retrospective nature of our study, we could not ascertain any prior use of these medications among our patients who sometimes may have visited other health facilities before presentation. These may account for the relative paucity of these signs in our study even in those with complicated appendicitis. Another reason may be clerical errors in documentation and storage of data.

Clinical features (symptoms and signs) are said to be reliably predictive of acute appendicitis in 83.4% of individuals [25] and are often discriminatory between uncomplicated and complicated cases. However, to improve the diagnostic yield, some clinicians added laboratory parameters like total while cell counts and differentials [7, 26], C-reactive protein [5, 6, 8], and serum bilirubin [9]. These however are not specific to the disease and can be elevated in other cases of acute inflammation. But when they are used as complimentary evidence, the diagnosis of acute appendicitis is thought to improve.

Similarly, medical imaging has been shown to improve diagnosis, especially in females in whom diseases of the ovaries, tubes, and right adnexa may mimic acute appendicitis. Thus, ultrasonography of the abdomen and pelvis in them is often performed in the diagnostic workup for acute appendicitis. Ultrasonography has been reported to have a sensitivity of 90%, and a specificity of over 90% in experienced hands [27]; but from our study, we found these to be 70.5% and 36.8% respectively. Ultrasonography is user dependent, and the appendix may not be easily visualized due to increased bowel gases: from inflammation-induced localized bowel distension. As a result, a computerized tomography scan, where accessible, has been considered to be superior to ultrasonography in the diagnostic workup for acute appendicitis [27]. However, this modality is associated with radiation exposure and may be unsafe in pregnant patients. In the latter group of individuals, magnetic resonance imaging (MRI) is considered an alternative study [27]. Our study had only ultrasonography and CT scan as preferred imaging modalities. There was no record of the use of MRI, and this could be a result of the presence of a dedicated maternal and child hospital (MCH) within the same city as our hospital. At the MCH, obstetrics, gynecological and pediatric diseases like appendicitis in pregnancy and children are managed. Based on our hospital policy, patients less than 14 years old and pregnant women are managed at the MCH, while those 14 years and above are managed in our hospital.

Our study found out that a CT scan was superior to an ultrasound scan in diagnosis. This conclusion was arrived at, based on the higher sensitivity rate and diagnostic odds ratio and its low false negative ratio associated with CT scans when compared with ultrasonography. The downside of the CT scan from this study was the relatively high false positive rate and low true negative values. A patient who had both USS and CT scan diagnosis of acute appendicitis was noted to have a histology report of a normal appendix. This supports the notion that both man and sometimes technology are not infallible. It is however noteworthy that of the 44 patients who had surgical management of their illness based solely on clinical diagnosis alone, appendicitis was confirmed in 42 (95.4%) of them. This, in a way, reinforces the belief that the diagnosis of acute appendicitis can be safely made on clinical grounds.

Traditionally, the management of acute appendicitis has been done by appendicectomy. However, in recent times, there has been advocacy for non-operative care of uncomplicated appendicitis in some selected cases [10, 11]. The latter position has not been globally accepted. Surgery for appendicitis could be done as an open or as a laparoscopic surgery technique. The laparoscopic approach is the current standard of care, and the open surgical approach is practiced in places where either the technology or the expertise for use of the technology is not available. In our center, both surgical approaches are practiced. For the open surgical approach, the grid-iron and the Lanz incisions are preferred for clinically uncomplicated cases, while midline incisions are reserved for those in whom doubts are entertained as to the diagnosis, or in whom diagnostic laparoscopy with the view to proceeding with the intervention was not possible. Although the Lanz incision gives a cosmetically acceptable scar, some surgeons in our study preferred the grid-iron incision as it is possible to extend it to improve surgical access. Also, some experienced surgeons could use it in some cases of complicated appendicitis encountered during the surgery. This preference for grid-iron incisions could explain the low rates of midline infra-umbilical incisions in this study. Our study did not reveal any complications or challenges associated with laparoscopic appendicectomy among the patients. This may not be unconnected with poor record keeping, and the paucity of data associated with a retrospective study like this one.

The limitations encountered with this study were principally limitations associated with retrospective studies generally. The record-keeping was poor and sometimes incomplete; and this was the reason for the non-inclusion of established clinical scores, like the Alvarado and the Raja Isteri Pengiran Anak Saleha Appendicitis (RIPASA) [11], in our study. Also, in our center, there are no standard guidelines on the investigative modality for patients with acute appendicitis, and this was the reason the role of laboratory tests (except complete blood count) was not fully explored. Similarly, during the study period, there was no established guideline on the use of medical imaging in patients with suspected acute appendicitis. This also could explain why either or both ultrasonography and CT scan were used in some patients but not in others: the indication and type of imaging done appear to be at the discretion of the admitting physician.

## Conclusions

This retrospective study evaluated some demography characteristics of patients who had appendicectomy in a tertiary hospital in Saudi Arabia. It reported common clinical features of acute appendicitis encountered in our practice, and the reliability of ultrasonography and CT scans in the diagnosis of the condition. We also reported that the diagnosis of acute appendicitis can still be based solely on clinical evaluation (symptoms and signs) in experienced hands. Furthermore, medical imaging techniques like ultrasonography and CT scan, though helpful in making diagnoses, can miss the diagnosis sometimes. Thus, they do not confer a zero percent negative appendicectomy rate when used individually or combined. Laparoscopic technique in appendectomy is popular, although the open approach was found to be most practiced in this study. Finally, while we would love to advocate that sound clinical evaluation in the management of diseases should be jettisoned due to the deployment of advancing technologies in medical practice, we feel that these technologies should instead complement good clinical acumen: this is what makes us clinicians and not robots.
